# Biomarkers to detect membranous nephropathy in Chinese patients

**DOI:** 10.18632/oncotarget.12014

**Published:** 2016-09-13

**Authors:** Li Lin, Wei Ming Wang, Xiao Xia Pan, Jing Xu, Chen Ni Gao, Wen Zhang, Hong Ren, Jing Yuan Xie, Pin Yan Shen, Yao Wen Xu, Li Yan Ni, Nan Chen

**Affiliations:** ^1^ Department of Nephrology, Institute of Nephrology, Ruijin Hospital, School of Medicine, Shanghai Jiaotong University, Shanghai, China

**Keywords:** chronic kidney disease, PLA2R, retinol binding protein, THSD7A, membranous nephropathy, Immunology and Microbiology Section, Immune response, Immunity

## Abstract

Anti-M-type phospholipase A_2_ receptor (anti-PLA_2_R) is a widely accepted biomarker for clinical idiopathic membranous neurophathy (IMN). However, its ability to differentiate between IMN and secondary MN (SMN) is controversial. The objective of this study was to assess clinical MN biomarkers in blood, tissue and urine samples from Chinese patients. In total, 195 MN patients and 70 patients with other glomerular diseases were prospectively enrolled in the study. Participants were followed up for average of 17 months (range 3-39 months). Anti-PLA_2_R and anti-THSD7A (thrombospondin type-1 domain-containing 7A) were detected only in MN patient sera and not in controls. Serum anti-THSD7A and THSD7A-positive biopsies were detected in 1/18 and 2/18 PLA_2_R-negative MN cases, respectively. PLA_2_R and THSD7A were detected in 72.27% and 40% of SMN cases, respectively. While serum positivity for both anti-PLA_2_R and anti-THSD7A at the time of renal biopsy was specific to MN patients, neither antigen could discriminate between primary and secondary MN. We also found that high urinary levels of retinol binding protein (RBP) predicted poor proteinuria outcomes in study participants. Patients with low or medium urinary RBP levels achieved remission more frequently than those with high RBP.

## INTRODUCTION

Membranous nephropathy (MN) is the most common cause of nephrotic syndrome (NS) in Caucasian adults, accounting for 30-40% of cases [1]. In China, MN rates are increasing rapidly in patients with primary glomerular nephropathy, from 7.1% in 2000 to 22.7% in 2009-2011 [2]. MN is twice as common in men as in women, with a median age of onset in the early 50s (39.64%), and is the most common primary glomerular disease in patients over 60 years of age. The prevalence of MN in children is relatively low, accounting for less than 5% of biopsy diagnoses [3].

MN diagnosis conventionally relies on kidney biopsy. Approximately 75% of cases are idiopathic (IMN), with the remainder secondary to infection (e.g. hepatitis B), systemic autoimmune disease (e.g. lupus), medications (e.g. NSAIDs) and certain malignancies [4-6]. An IMN diagnosis is made by excluding secondary causes according to history, physical examination and appropriate laboratory tests, and by careful microscopic examination of the kidney biopsy. Previous studies showed that circulating autoantibodies against the M-type phospholipase A_2_ receptor (anti-PLA_2_R) are detectable in 52-82% of IMN patients and are absent or very uncommon in patients with secondary MN (SMN) [7-17]. As a result, anti-PLA_2_R has been widely accepted as a biomarker for clinical IMN diagnosis due to its high sensitivity and specificity. However, anti-PLA_2_R might be detectable in certain SMN cases, such as tumor- and sarcoidosis-associated MN [10, 18]. In 2014, thrombospondin type-1 domain-containing 7A (THSD7A), another antigen similar to PLA_2_R in structure, was identified as a second potential IMN biomarker [19]. Approximately 8-14% of anti-PLA_2_R seronegative patients had autoantibodies against THSD7A (anti-THSD7A) [19-21]. The incidence of THSD7A-related MN was 13.63% in a European cohort and 8.18% in a Boston cohort in PLA_2_R-unrelated IMN [19]. Larsen reported a 3% incidence of THSD7A-positive glomeruli in MN in the United States [21]. Iwakura reported this as 9.1% in Japanese [20]. However, the usefulness of PLA_2_R and THSD7A has not yet been assessed in Chinese MN patients. In addition, the prognostic values of low molecular weight (LMW) proteins in MN patient urine must be validated in different populations [22-24].

Here, we report anti-PLA_2_R and anti-THSD7A serum levels, and PLA_2_R and THSD7A staining in glomeruli of MN patients. We compared clinical parameters, pathological features and clinical outcomes between PLA_2_R-positive and -negative MN cohorts. Moreover, this study evaluated the utility of urinary LMW proteins as predictors of proteinuria outcome. To relate clinical characteristics to baseline anti-PLA_2_R and anti-THSD7A levels at the time of renal biopsy, and to prove whether the titer of anti-PLA_2_R, urinary LMW proteins, including urinary β2 microglobulin (β2 m), urinaryα1 microglobulin(α1m), the urinary retinol binding protein (RBP) and urinary N-Acetyl—β-D-indiglucosaminidase (NAG) affect disease outcome. This is the first study to objectively assess the diagnostic and prognostic values of blood, urine and kidney biomarkers in Chinese MN patients.

## RESULTS

### PLA_2_R in MN diagnosis

**Figure 1:**
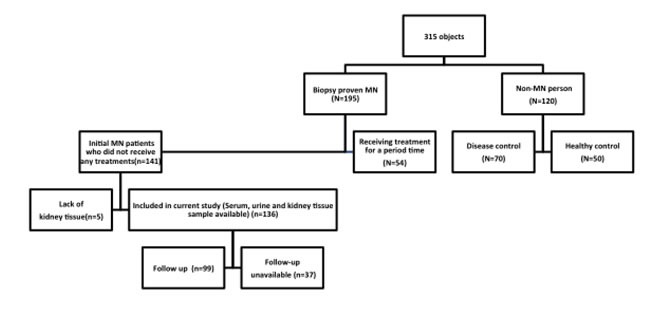
Patient recruitment parameters and inclusion flowchart

**Figure 2:**
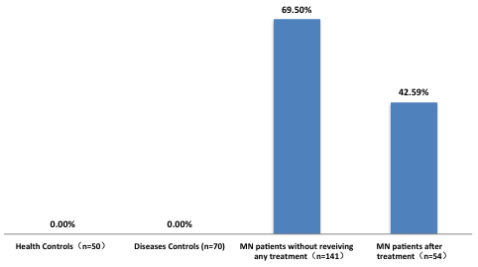
Rates of serum anti-PLA_2_R positivity in patients and controls Disease controls: IgA nephropathy (*n* = 5), minimal change disease (*n* = 11), focal segmental glomerular sclerosis (*n* = 10), membrano-proliferative glomerulonephritis (*n* = 6), lupus nephropathy (*n* = 21, including 1 LN-III, 2 LN-IV, 1 LN-V, 10 LN-IV+V, 7 LN-III+V), ANCA-associated vasculitis (*n* = 5), amyloidosis (*n* = 3), lymphoma (*n* = 1), Goodpasture syndrome (*n* = 1), diabetic nephropathy (*n* = 1) and ANA(autoantibody to nuclear antigen) (+) of unknown significance (*n* = 50).

A total of 315 subjects were recruited for this study, including 195 renal biopsy-confirmed MN patients. Serum samples were collected from 54 of these patients cross-sectionally after treatments (Figure 1). The remaining 141 patients did not receive any MN-specific treatments. We also tested 70 patients with non-MN diseases and 50 healthy controls. Anti-PLA_2_R and anti-THSD7A were only found in patients with MN, and were not detected in either healthy controls or non-MN patients (Figure 2 & S1). Among the non-MN patients, 18 out of 21 lupus nephropathy (LN) patients with class V disease (1 LN-V, 10 LN-IV+V, 7 LN-III+V) were negative for circulating anti-PLA_2_R/anti-THSD7A and glomeruli PLA_2_R/THSD7A.

Of the 54 MN patients whose serum was sampled cross-sectionally, 23 had circulating anti-PLA2R antibodies. Anti-PLA_2_R was detected in 8.33% of complete remission (CR) patients, 31.58% of partial remission (PR) patients and 69.57% of no remission patients (*P* = 0.001) (Table S1, Figure S3).

**Figure 3:**
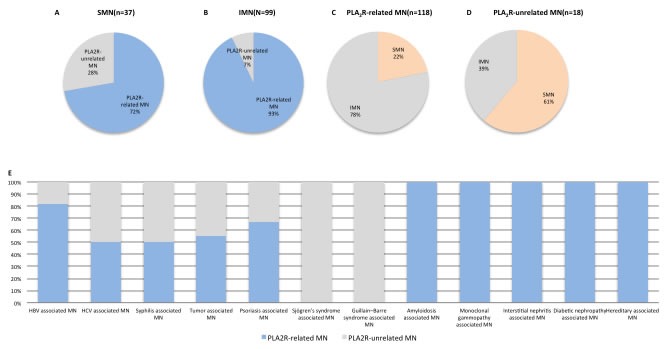
PLA_2_R in SMN PLA_2_R positivity rates in SMN A. and IMN B. SMN rate in PLA_2_R-related MN C. and PLA_2_R-unrelated MN D. PLA_2_R-related MN in SMN E. Secondary MN associations: hepatitis B, *n* = 11; hepatitis C, *n* = 1; syphilis, *n* = 2; tumor, *n* = 8; psoriasis, *n* = 3; Sjögren's syndrome, *n* = 2; Guillain-Barre syndrome, *n* = 1; amyloidosis, *n* = 1; monoclonal gammopathy, *n* = 1; interstitial nephritis, *n* = 2; diabetic nephropathy, *n* = 2; hereditary MN, *n* = 2; hepatitis B and tumor, *n* = 1. Tumor-associated MN: Laryngocarcinoma, *n* = 1; lymphoma *n* = 1; rectal carcinoma, *n* = 1; mediastinal tumor, *n* = 1; breast cancer, *n* = 1; lung cancer, *n* = 1; thyroid tumor, *n* = 1; gynecological cancer, *n* = 1.

Of the patients who were followed up longitudinally (*n* = 136), the majority of PLA_2_R-related MN patients were men (58.87%), aged 15 to 83 (median = 39.8). Positive serum anti-PLA_2_R and glomerular PLA_2_R rates were 69.50% and 83.21%, respectively, in this population (Figure S1 & S2). The rate of PLA_2_R-related MN was 87.23%. In 37 SMN cases, 72.27% were PLA_2_R-related MN (Figure 3A), and the anti-PLA_2_R titer was 217±291 RU/ml (Table 1). In the remaining 99 patients, the rate of PLA_2_R-related MN was 92.92% (Figure 3B), and the anti-PLA_2_R titer was 154±255 RU/ml (Table 1). More SMN patients exhibited PLA_2_R-unrelated than PLA_2_R-related MN (61.11% vs 21.13% *P* = 0.01) (Figure 3C-3D). 56% of tumor-associated MN was PLA_2_R-related. 50-82% of MN secondary to infection (HBV-, HCV- and syphilis-associated SMN) was PLA_2_R-related. PLA_2_R was also detected in other SMN patients, including those with psoriasis-, monoclonal gammopathy-, interstitial nephritis-, diabetes nephropathy- and hereditary-associated MN (Figure 3E, Table 1).

**Table 1:** PLA2R-related and -unrelated MN in SMN

	Hepatitis B (*N*=11)	Hepatitis C (*N*=1)	Syphilis (*N*=2)	Tumor (*N*=9)	Psoriasis (*N*=3)	Sjögren's syndrome (*N*=2)	Guillain— Barre syndrome (*N*=1)	Amyloidosis (*N*=1)	Monoclonal gammopathy (*N*=1)	Interstitial nephritis (*N*=3)	DN (*N*=2)	Familial (*N*=2)	IMN (*N*=99)	Type V LN (*N*=18)
PLA2R-related MN(*n*=118)	9	1	1	5	2	0	0	1	1	3	2	2	92	0
Anti-PLA_2_R(+) by IFA (n)	9	1	1	3	2	0	0	1	1	3	2	2	71	0
Average titer of anti-PLA_2_R (RU/ml)	241	184	200	204	85	0	/	400	41	598	123	329	154	/
PLAR(+) in glomeruli (n)	8	1	1	5	2	0	0	1	1	3	2	2	89	0
PLA_2_R-unrelated MN(*n*=18)	2	/	1	4	1	2	1	/	/	/	/	/	7	18
Average titer of anti-PLA_2_R (RU/ml)	<0	/	0	1	6	1	0	/	/	/	/	/	0.1	/

18 patients with class V LN included: LN-V (n=1), LN-IV+V (n=10), LN-III+V (n=7).

Patients with circulating anti-PLA_2_R and with PLA_2_R deposits in glomeruli were included in group 1, patients negative for circulating anti-PLA_2_R, but with PLA_2_R in glomerular deposits were included in group 2, patients positive for circulating anti-PLA_2_R, but negative for PLA_2_R in glomerular deposits were included in group 3 and patients negative for both circulating anti-PLA_2_R antibodies and PLA_2_R in glomeruli were included in group 4. There were 89 (65.44%), 24 (17.64%), 5(3.68%) and 18 (13.24%) patients in groups 1, 2, 3 and 4, respectively (Table 2). Cases in the group 1 had a mean 24-h proteinuria of 5.43g. This was higher than 3.52g, the mean 24-h proteinuria level for cases in the group 2. There was no statistical difference in sex, age, time at renal biopsy from onset or follow-up time between PLA_2_R-related and PLA_2_R -unrelated MN patients. Furthermore, there was no significant difference in any relevant clinical parameters, such as serum creatinine (Scr) or glomerular filtration rate (GFR), among the four groups. More anti-PLA_2_R-positive patients developed nephrotic-range proteinuria (80.90%) than did anti-PLA_2_R-negative patients (50.00%, *P* = 0.003). All 136 MN biopsies were analyzed for the extent of tubulointerstitial fibrosis and glomerular lesions. Interstitial fibrosis was found in 117 of 136 specimens. There were no significant differences in the percentage of patients with interstitial fibrosis, glomerular lesions or segmental glomerulosclerosis among the four groups (Table 3).

**Table 2:** Clinical features, baseline PLA_2_R levels and outcomes in PLA2R-related and -unrelated MN

Clinical features	GROUP1(PLA_2_R + anti-PLA_2_R +)	GROUP2(PLA_2_R + anti-PLA_2_R -)	GROUP3(PLA_2_R - anti-PLAR +)	GROUP4(PLA_2_R - anti-PLA_2_R -)		
					GROUP1-4	GROUP1 and 2
Index at baseline (n=136)						
Number of patients	89(65.44)	24(17.64)	5(3.68)	18(13.24)	/	/
Sex (M%)	52(58.42)	13(58.33)	3(60.00)	11(61.11)	0.972	0.441
Age (year)	59(15-83)	54.5(23-77)	46(25-66)	55(17-79)	0.524	0.276
Anti-PLA_2_R assay from on set (month)	2(0.25-180)	1.5(0.25-12)	2(1-24)	3.5(0.25-12)	0.284	0.135
IS use before assay(%)	4(4.49)	0(0)	0(0)	1(5.56)	0.693	0.379
24h-Proteinuria (g/24)	5.43(0.59-21.02)	3.52(0.49-6.92)	12.33(5.02-25.78)	5.00(0.68-22.98)	0.001*	0.001*
Albumin (g/l)	21(10-39)	24(16-35)	14(13-23)	24(11-35)	0.022*	0.042*
Serum creatinine (umol/l)	68(23-262)	65(42-120)	63(58-97)	75.5(51-160)	0.473	0.234
GFR(ml/min/1.73m^2^)	108(22-151)	114(57-150)	113(71-131)	104(38-135)	0.473	0.131
Nephrotic syndrome (%)	72(80.90)	12 (50)	5 (100)	13(72.22)	0.01*	0.003*
Follow-up(month)	17(3-39)	14 (3-39)	28 (8-34)	19 (3-33)	0.423	0.355
Index of outcome (*n*=99)						
Number of patients	68	13	4	14	/	/
Disease active(%)	17(25.00)	2( 15.38)	1(25)	1(7.15)	0.687	0.07
Partial remission(%)	27(39.71)	6 (46.16)	2(50)	5(35.71)		
Completely remission(%)	24(35.29)	5( 38.46)	1(25)	8( 57.14)		
Time to PR(week)	2 (0.25-18)	2.5 (0.25-15)	4(2-4.25)	4 (1-21)	0.305	0.258
GFR >25%	2(2.25)	0	0	0	/	/

Group 1, serum anti- PLA_2_R - and glomerular PLA_2_R -positive patients; Group 2, glomerular PLA_2_R -positive but serum anti- PLA_2_R -negative patients; Group 3, serum anti- PLA_2_R -positive but glomerular PLA_2_R -negative patients; and Group 4, serum anti- PLA_2_R -negative and glomerular PLA_2_R -negative patients.

IS: Immunosuppressor.

* *P* value <0.05 was considered as statistical significance.

**Table 3:** Renal biopsy findings (n=136)

Histologic characteristics	GROUP1(PLA_2_R + anti-PLA_2_R +)	GROUP2(PLA_2_R + anti-PLA_2_R-)	GROUP3(PLA_2_R - anti-PLA_2_R +)	GROUP4(PLA_2_R - anti-PLA_2_R -)	*P*value
Number of patients	89	24	5	18	
Glomerular lesions (electron microscopy) %					
I(%)	19 (22.09)	7(19.17)	0 (0)	6 (33.33)	0.795
II(%)	52 (60.47)	14 (58.33)	4 (80)	9 (50)	
III(%)	14 (16.28)	3 (12.50)	1 (20)	3 (16.67)	
Interstitial fibrosis (%)					
None	13 (14.94)	5 (20.83)	0 (0)	1 (5.56)	0.840
Minor	75 (84.27)	19 (79 .17)	5(100)	13 (94.44)	
Moderate	1 (1.12)	0 (0)	0 (0)	0 (0)	
Focal segmental sclerosis	11 (12.36)	1 (4.17)	1 (20)	1 (5.56)	0.506
IgG4 + (%)	100	100	100	55.56	<0.001

Interstitial fibrosis minor :< 25% of the tubulointerstitial space involved, moderate :> 25% but < 50% of the tubulointerstitial space involved, extensive :> 50% of the tubulointerstitial space involved

### THSD7A in MN diagnosis

**Figure 4:**
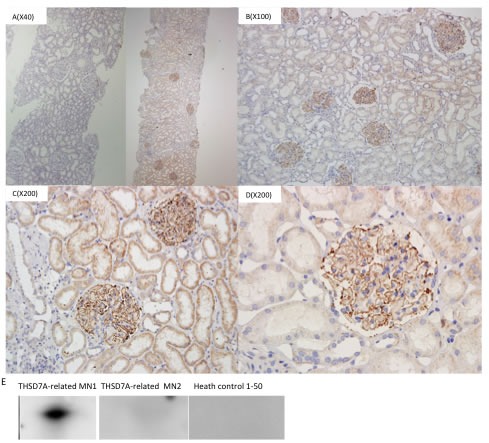
THSD7A in glomeruli and serum Left: THASD7A negative glomeruli. Right: Strong global granular capillary loop THASD7A staining. Magnification ×40 A., ×100 B., ×200 C. and ×400 D. Western blotting to detect THSD7A in patient serum E.

Of 136 patients, 2 patients stained positive for glomerular THSD7A via immunohistochemical analysis (Figure 4A-4D). Serum anti-THSD7A was detected in one of these patients via western blotting (Figure 4E). Both of the THSD7A-related MN patients were negative for glomerular PLA_2_R and serum anti-PLA_2_R. No patient exhibited dual positivity for PLA_2_R and THSD7A.

In THSD7A-related MN, the ratio of females to males was 0.667. Patient ages ranged from 24 to 62, and 40% had nephrotic syndrome at the time of renal biopsy (Table 4). 40% of THSD7A-related MN was secondary to thyroid tumor or HBV infection. Histologic and biochemical THSD7A-related MN characteristics resembled those of PLA_2_R-related MN. During follow-up (2-24 months), 80% of THSD7A-related MN patients achieved remission (CR or PR)(Table 4).

**Table 4:** Clinical features at baseline and outcome in THSD7A-related MN

	Clinical features			THSD7A		Histologic characteristics			Biochemical index at baseline				Index of outcome	
Number	Gender	Age(yr)	SMN or IMN	Serum anti-THSD7A	Glomeruli THSD7A	Glomerular lesions	Interstitial fibrosis	Glomeluli IgG4	24h-proteinuria (g/24h)	Alb (g/l)	Scr (mmol/L)	GFR (ml/min/1.73m^2^)	Follow-up (month)	proteinuria outcome
Patient 1	Male	46	tumor associated MN	-	+	MN-I	Minor	+	6.356	18	63	126.40	24	CR
Patient 2	Male	62	HBV associated MN	+	+	MN-1	Minor	+	5.619	19	65	114.75	13	NR
Patient 3	Female	35	IMN	+	+	MN-II	Minor	+	1.482	28	53	137.54	8	CR
Patient 4	Female	24	IMN	-	+	MN-II	Minor	+	2.993	24	50	120.68	3	CR
Patient 5	Female	32	IMN	+	+	MN-II	Minor	+	2.819	32	61	120	4	PR

Patient 1 and patient 2 from this study, patients 3-5 from the following study

### PLA_2_R as a prognostic biomarker for MN

99 patients remained at the end of the follow-up period and were classified as in remission (*n* = 78) or no remission (*n* = 21). Follow-up times for these two groups averaged 17 (3-39) and 13 months (3-36), respectively (*P* = 0.154). During the study, only two patients had an increase in Scr by ≥ 25% and GFR ≤ 60 ml/min/1.73m^2^.

In group 1, 17 (25%) patients still had active disease at the end of the follow-up period, 24 (35.29%) were in CR, and 27 (39.71%) were in PR. In group 2, 6 (46.16%) patients were in PR and 5 (38.46%) were in CR. In group 3, 1 (25%) patient was in CR and 2 (50%) were in PR. In group 4, 8 (57.14%) patients were in CR and 5 (35.71%) were in PR (Table 1). Remission rate was the same among the four groups (*P* = 0.687), and the time to achieving PR in anti-PLA_2_R-positive patients was similar to that of anti-PLA_2_R-negative patients (*P* = 0.258) (Table 2).

**Figure 5:**
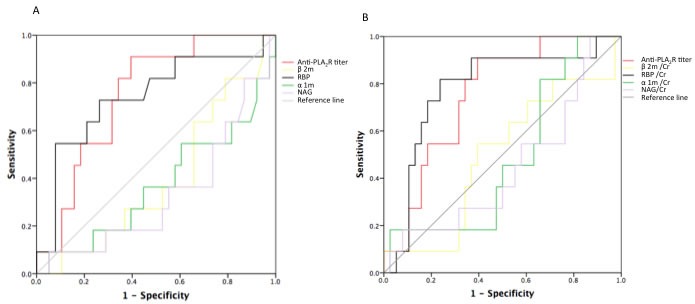
ROC curves for urinary excretion rates of LMW proteins and serum anti-PLA2R titer AUCs for predicting no remission were as follows: anit-PLA_2_R: 0.751 (0.608-0.895), *p* = 0.012; RBP: 0.740 (0.559-0.922), *p* = 0.016; α1m: 0.370; β2m: 0.394; NAG: 0.331 A. AUCs corrected for urinary creatinine were as follows: RBP/Cr: 0.778 (0.616-0.939), *p* = 0.005; α1m/Cr: 0.483; β2m/Cr: 0.495; NAG/Cr: 0.445 B.

The baseline anti-PLA_2_R titer in the no remission group was much higher than in the remission group (*P* = 0.0035). The anti-PLA_2_R titer AUC (area under ROC curve) was 0.751 when anti-PLA_2_R titer at the time of renal biopsy was assessed as a predictor of no remission (Figure 5). Patients were divided into three groups based on anit-PLA_2_R titer. Anti-PLA_2_R levels < 29.76 RU/ml were included in tertile 1 (low titer), 29.76-98.82 RU/ml in tertile 2 (medium titer) and > 98.829 RU/ml tertile 3 (high titer). There was no difference in remission rate among the three groups (*P* = 0.130). A Cox model indicated that baseline anti-PLA_2_R level was not associated with disease activity, adjusting for the effects of all other variables.

### LMW proteins as biomarkers to predict MN prognosis

When assessing urinary LMW proteins as predictors of no remission, urinary α1 m, β2 m, RBP and NAG AUCs were 0.370, 0.394, 0.740 and 0.331, respectively (Figure 5A). When corrected by urinary creatinine (Cr), ROCs were as follows: α1 m/Cr 0.483, β2 m/Cr 0.495, RBP/Cr 0.778, NAG/Cr 0.445 (Figure 5B). Patients were divided into three groups based on baseline RBP/Cr. 38.71% of patients in the high RBP/Cr group had not achieved remission by the end of the follow-up period, while only 18.74% and 6.24% of patients in the low and medium level groups, respectively, had not achieved remission (*P* = 0.003) (Table 5).

**Table 5:** Disease status at the end of follow-up in three groups divided by RBP/creatinine (mg/mmol) at baseline

Group	Duration(month)	Complete remission(%)	Partial remission(%)	No remission(%)
Low level(<0.1941)	14(3-39)	40.63	40.63	18.74
Medium level(0.1941-0.4772)	16(3-36)	34.38	59.38	6.24
High level(>0.4772)	18(3-36)	45.16	16.13	38.71
*P* value	0.659	0.003		

## DISCUSSION

This prospective study assessed Chinese patient blood, kidney tissue and urine samples to identify new MN biomarkers for clinical application, and to assess existing biomarkers. Our results showed that serum anti-PLA_2_R and anti-THSD7A specificities for MN diagnosis were both 100%, and sensitivities were 42.59-69.5% and 1.47%, respectively. As a non- inflammatory autoimmune disease, MN affected affecteds the kidney glomerulus, resulting in the formation of immune deposits on the outer aspect of the glomerular basement membrane [1]. The binding of circulating antibodies specific for an intrinsic antigen presented on the basal surface of the podocytes is the mechanism at play in most forms of adult MN [25]. THSD7A and PLA_2_R exhibit similar structural and biochemical properties [19, 21, 26]. These two antigens are both located in podocytes, and autoantibodies against both are predominantly classified into the IgG4 subclass [7, 19]. A number of molecular biological and genetics studies have shown that PLA_2_R plays an important role in MN [8-11, 13, 27-30]. The dominant humoral epitope is in the N-terminal region of PLA_2_R and is recognized by 90% of human anti-PLA2R autoantibodies [15, 31]. Moreover, the epitope (9-amino acid peptide within the 31-mer) itself is capable of inhibiting 47% of binding shares homology with a bacterial enzyme, thus invoking molecular mimicry as a potential initiator of autoimmunity [31]. In addition, genome-wide association studies (GWAS) revealed significant associations between the 6p21 HLA- DQA1 and 2q24 PLA_2_R loci with MN in European patients [29], which was confirmed in populations from Asia [30]. Because PLA_2_R and THSD7A play an essential role in the pathogenesis of MN, the rates of anti-PLA_2_R and anti-THSD7A positivity were significantly higher in MN patients than in other series. Consequently, in elderly patients and in patients in poor clinical condition or experiencing life-threatening complications, such as lung thrombus, kidney biopsy might be replaced with serological anti-PLA_2_R and anti-THSD7A detection for diagnosis of MN. We found that 17.64% patients had no circulating anti-PLA_2_R, although PLA_2_R was detected in glomerular immune deposits. Thus, the absence of circulating PLA_2_R at the time of kidney biopsy did not rule out a diagnosis of PLA_2_R-related MN. We also identified five patients with circulating anti-PLA_2_R, but without detectable PLA_2_R in glomerular deposits.

We found that PLA_2_R and THSD7A detection discriminated MN from other nephropathies, but could not distinguish secondary from primary MN. SMN in this study included patients with infections (hepatitis B, hepatitis C, syphilis), tumors, autoimmune diseases (psoriasis, Guillain-Barre syndrome), monoclonal gammopathy and interstitial nephritis. Lupus-associated MN patients were always PLA_2_R negative. In SMN, the PLA_2_R positivity rate was 72.97%; thus, PLA_2_R in glomeruli and anti-PLA_2_R in circulation are not suitable for distinguishing IMN from SMN. Previous studies reported anti-PLA_2_R positivity in SMN cases in patients with HBV, lupus, tumors and. sarcoidosis [10,14,18,32]. One interpretation of these findings is that these patients had primary PLA2R-related MN causally unrelated to their additional pathologies. A second interpretation is that these systemic disease processes may activate the immune system to stimulate autoantibody production against PLA_2_R. Therefore, whether or not PLA_2_R can be used to differentiate IMN from SMN remains controversial.

Several recent studies correlated anti-PLA_2_R levels with clinical manifestations of disease status, specifically, decreasing anti-PLA_2_R during remission and increasing levels during relapse [12, 13, 17,33-35]. We found that anti-PLA_2_R positive rates in patients with no remission were significant higher than in patients with CR or PR. Moreover, patients positive for anti-PLA_2_R exhibited more severe proteinuria and lower albumin levels in contrast to patients negative for anti-PLA_2_R at MN onset. Although baseline anti-PLA_2_R titers were lower in the remission group compared to no remission, the anti-PLA_2_R titer AUC for predicting no remission was 0.751. There was no difference in remission rates between anti-PLA_2_R-negative and -positive patients during follow-up. Similarly, remission rates were the same among patients with low, medium and high baseline anti-PLA_2_R titers. Thus, anti-PLA_2_R levels dynamically reflected disease status, but anti-PLA_2_R application as a predictive biomarker requires additional verification.

This study found that high urinary RBP levels might predict poor proteinuria outcome. AUC of urinary RBP/Cr was 0.778, which was higher than that of anti-PLA_2_R titer. More patients with low or medium urinary RBP levels achieved remission as compared to those with high RBP. Another study reported that LMW urinary proteins predicted kidney function outcomes in 129 MN patients [22]. AUCs were 0.81 and 0.81 for β2m and α1m, respectively, to predict a GFR increase of 25% from baseline.

Although anti-PLA_2_R titer may also be an early indicator of risk of progressive renal function loss [12, 13, 17, 26-28], in our study only two (1.42%) patients showed an increase of > 25% baseline Scr and GFR reaching < 60 ml/min/1.73 m^2^ during follow-up. Since assessing renal function changes requires a longer period of observation time, we did not analyze correlations between biomarkers and loss of renal function. Our findings were limited by a relatively small study sample size and single-center patient recruitment. Besides, the observation time was relatively short. Further research with larger sample sizes and longer follow-up times are needed to validate PLA_2_R and urinary RBP sensitivities and utilities in diagnosing MN.

In conclusion, we confirmed that anti-PLA_2_R and anti-THSD7A were specifically detected in MN patient sera. Detection of PLA_2_R/THSD7A in kidney biopsies or of circulating anti-PLA_2_R/anti-THSD7A can discriminate between MN and LN, but not between IMN and SMN. As a predictive index for proteinuria outcome, urinary RBP might be superior to serum anti-PLA_2_R.

## MATERIALS AND METHODS

### Patients and tissue samples

In total, 315 patients were recruited between July 2012 and July 2014 from the Department of Nephrology, Ruijin Hospital, including 195 MN patients with confirmed renal biopsies, 70 non-MN nephrotic syndrome patients and 50 healthy controls. Serum samples from all 195 MN patients were tested for anti-PLA2R. Serum samples were collected cross-sectionally after treatments from 54 of these patients (Figure 1). Five of the 54 received ACEI/ARB (angiotensin converting enzyme inhibitors/angiotensin receptor blocker), two received steroid therapy, and 47 received steroids combined with immunosuppressive therapy (24 received cyclophosphamide, nine received cyclosporine, six received FK506, and eight received multi-target therapy) for varying lengths of time (1-123 months). The remaining 141 patients did not receive any MN-specific treatments. Serum and urine samples were collected longitudinally from these patients at the time of renal biopsy, before any invasive treatments, and were followed up at 3, 6, 9, 12 and 30 months. Follow-up tests included 24-h proteinuria, serum albumin (Alb), serum creatinine (Scr) and other biochemical indicators. Among 136 MN patients whose serum, urine and kidney tissue samples were available, 37 suffered SMN associated with hepatitis B (HBV), *n* = 11; hepatitis C (HCV), *n* = 1; syphilis, *n* = 2; tumor MN, *n* = 8; psoriasis, *n* = 3; Sjögren's syndrome, *n* = 2; Guillain-Barre syndrome, *n* = 1; amyloidosis, *n* = 1; monoclonal gammopathy, *n* = 1; interstitial nephritis, *n* = 2; diabetic nephropathy, *n* = 2; hereditary MN, *n* = 2; and hepatitis B and tumor associated MN, *n* = 1. We also enrolled 18 class V LN patients (LN-V, *n* = 1; LN-IV+V, *n* = 10; LN-III+V, *n* = 7).

### Study design

PLA_2_R-related MN was defined as being either positive for serum anti-PLA_2_R or glomerular PLA_2_R in biopsy-diagnosed MN. Similarly, THSD7A-related MN was defined as being either positive for serum anti-THSD7A or glomerular THSD7A in biopsy-diagnosed MN. Patients were divided into four groups depending on the presence of PLA_2_R antigen or its antibodies at the beginning of the study (Table 1). Presence of both circulating anti-PLA_2_R and glomerular PLA_2_R deposits was defined as group 1, presence of only glomerular PLA_2_R deposits or only circulating anti- PLA_2_R was defined as groups 2 and 3, respectively, and negative for both was defined as group 4. Clinical characteristics such as 24-h proteinuria, album, Scr, remission rate, etc. at baseline and follow-up were compared among the four groups. Patients were divided into two groups according to disease status at the end of follow-up: remission and no remission. Urinary LMW proteins, including βnm, urinary rinarurinary RBP and urinary NAG, were compared between the two groups to assess the specificity and sensitivity of urinary biomarkers, measured at the time of renal biopsy, in predicting proteinuria prognosis. All participants were fully consenting adults, and the IRB board of Ruijin Hospital approved this study.

Antibody prevalence was calculated for each group and clinical parameters were compared between patients with and without anti-PLA_2_R antibodies. 24-h proteinuria and Scr were measured every three months. The study end point was defined as CR (complete remission) or PR (partial remission). PR was defined as stable renal function (< 25% increase in GFR calculated by modification of diet in renal disease equation) with a 24-h proteinuria of 0.3-3.5 g and a proteinuria reduction ≥ 50% compared to baseline. CR was defined as a 24-h proteinuria < 0.3 g with stable renal function. Active disease was defined as a 24-h proteinuria > 3.5 g, persistence of > 50% baseline proteinuria or > 25% increase in Scr.

### Serum anti-PLA_2_R and anti-THSD7A measurements

Serum anti-PLA_2_R was assessed via indirect fluorescent antibody test (IFA) (FA1254-1005-50; EUROIMMUN AG, Lübeck, Germany). Serum was diluted 1:10 and anti-PLA_2_R detection was performed on an IFA Mosaic slide following the standard protocol. Serum anti-PLA_2_R titer was assessed via ELISA (Euroimmun, Lübeck, Germany; linearity: lower limit of detection 0.6RU/ml). A titer above 20 RU/ml was defined anti-PLA2R positive. Serum nti-THSD7A was detected by western blot. Recombinant THSD7A (Atlas Antibodies AB, Stockholm, Sweden) was electrophoresed under non-reducing conditions and proteins were transferred to nitrocellulose membranes according to standard protocols. Blots were blocked with 10% milk in Tris-buffered normal saline-Tween 20 (TBS-T). For the primary antibody, patient serum was diluted at a 1:100 ratio and incubated with blots overnight at 4°C. Peroxidase-conjugated donkey anti-human IgG (Euroimmun, Lübeck, Germany) was used as the secondary antibody at 4°C. Blots were incubated in chemiluminescent substrate, exposed to photographic film for 5 s to 2 min and developed in a Kodak X-Omat developer.

### Histological measurements

Renal biopsies were incubated with a rabbit polyclonal anti-PLA_2_R antibody (Atlas Antibodies AB, Stockholm, Sweden). PLA_2_R staining quality was evaluated by standard immunofluorescence microscopy. The presence of granular capillary loop staining in glomeruli was defined as positive, and the absence was negative. Each section was scored on a scale of 0-3+. A positive THSD7A immunohistochemical result was defined as strong diffuse global granular staining along capillary loops [21]. Interstitial fibrosis was assessed categorically as minor, moderate or extensive. Histological results were confirmed by two pathologists independently.

### Urinary measurements

Urinary α1m levels were measured by rate scatter nephelometry on a BN II automatic protein analyzer nephelometer (Siemens, Germany), urinary β2m by chemiluminescence on an Immulite2000 immunoassay system (Siemens, Germany), urinary RBP by Immune turbidimetry on a UniCel DxC800 Access immunoassay system (Beckman, USA) and urinary NAG by colorimetry on a UniCel DxC800 (Beckman, USA).

### Statistical analysis

For descriptive statistics, data were presented as means (± standard deviation) or medians (range) as appropriate. Kruskall-Wallis tests were used for comparisons among the four groups classified by serum anti-PLA_2_R and glomerular PLA_2_R levels. Proportions were compared by Chi-square analysis. Time to remission was assessed using the Kaplan-Meier method. A multivariate Cox regression analysis was conducted to identify potential risk factors associated with the development of active disease in this patient cohort. Clinical factors, including age, sex, PLA_2_R-related MN, anti-PLA_2_R levels, proteinuria, serum albumin, Scr, cholesterol, triglycerides, glomerular lesions and interstitial fibrosis at renal biopsy were adjusted in the Cox model. Correlations were assessed via Spearman Rank test. Receiver operating characteristic (ROC) curve was used to determine cut-off values for urinary predictors of proteinuria remission. All statistical analyses were performed using SPSS (version 17.0, SPPS Inc., Chicago, IL, USA). P < 0.05 was considered statistically significant.

## SUPPLEMENTARY MATERIALS FIGURES AND TABLES


