# Supplementary material for: Biomarkers to detect membranous nephropathy in Chinese patients
Source: Oncotarget. 2016 Sep 13;7(42):67868–79. doi: 10.18632/oncotarget.12014 (PMC5356526; doi:10.18632/oncotarget.12014)
Supplement: Supplementary file 1 [file oncotarget-07-67868-s001.pdf]

Biomarkers to detect membranous nephropathy in Chinese patients

Supplementary Material

Figure S1: The positive and negative feature of serum anti-PLA_2R by IFA. (A) Positive feature of anti-PLA2R by IFA **(B)** negative for anti-PLA2R

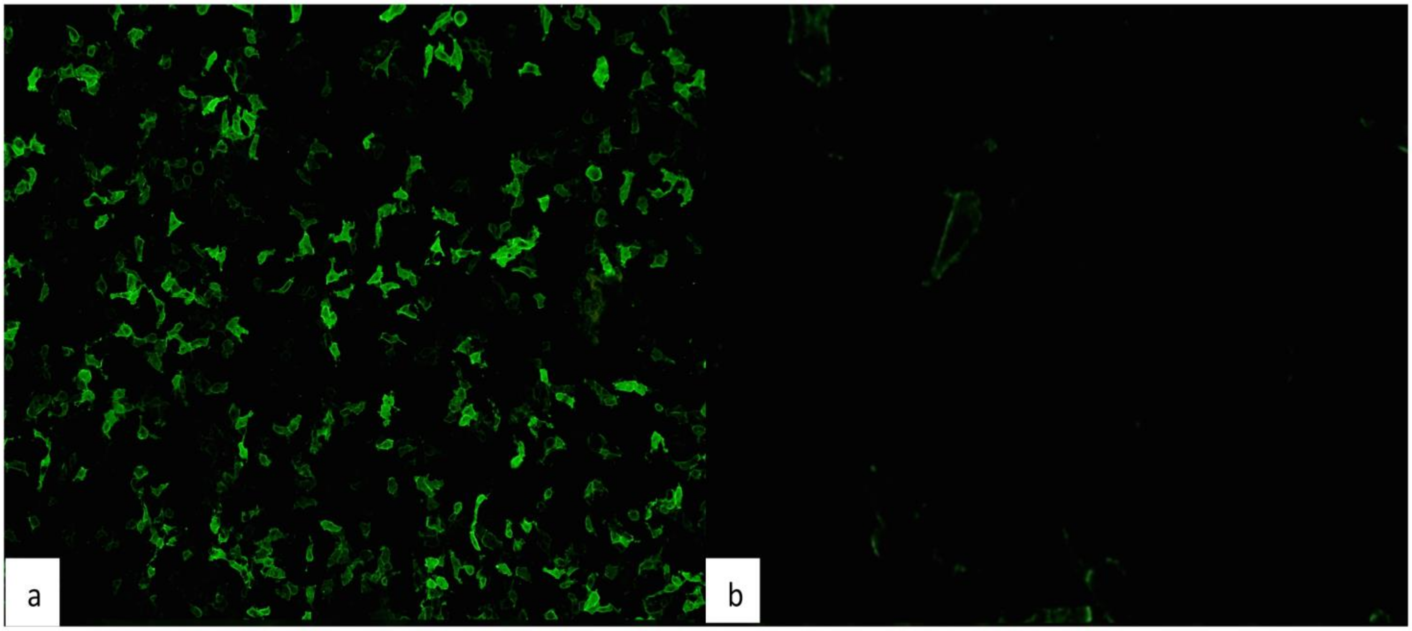

Figure S2: The positive and negative feature of glomeruli PLA2R antigen. Left: positive for PLA2R in glomeruli, Right: negative for PLA2R in glomeruli.

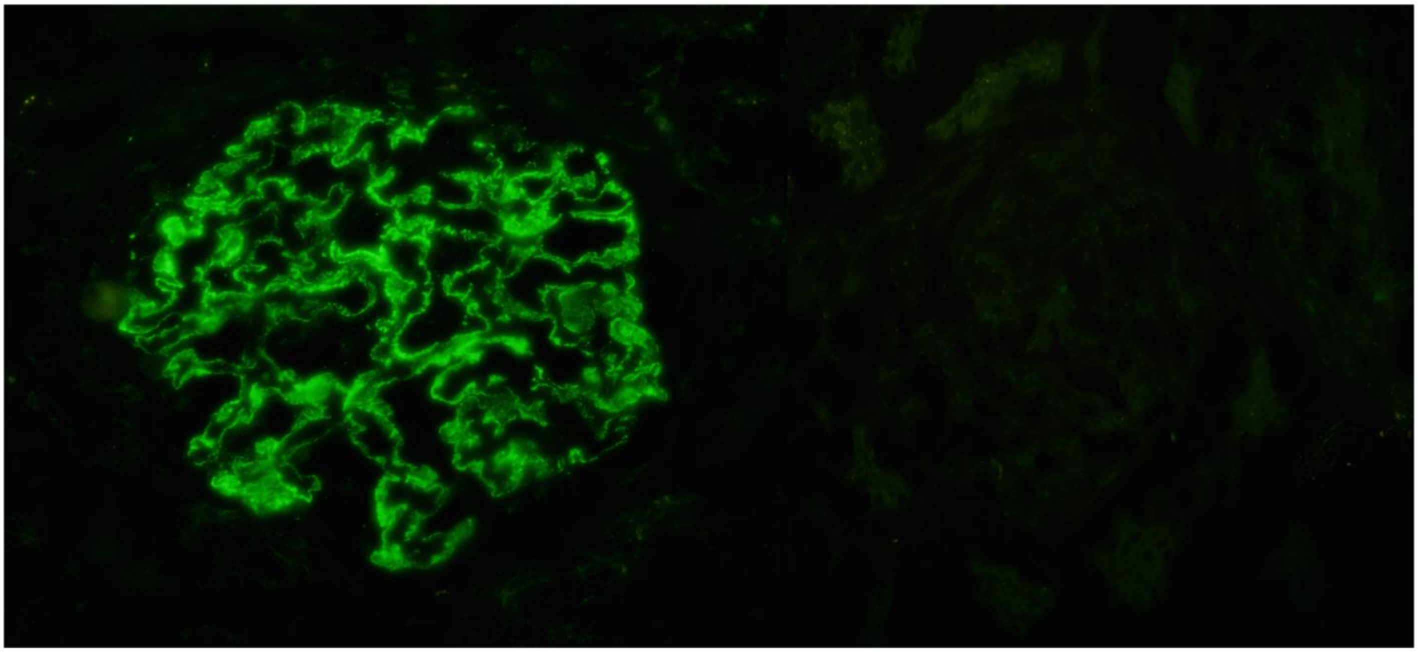

Figure S3: Positive rate of anti-PLA2R in MN in cross-sectionally group (n=54)

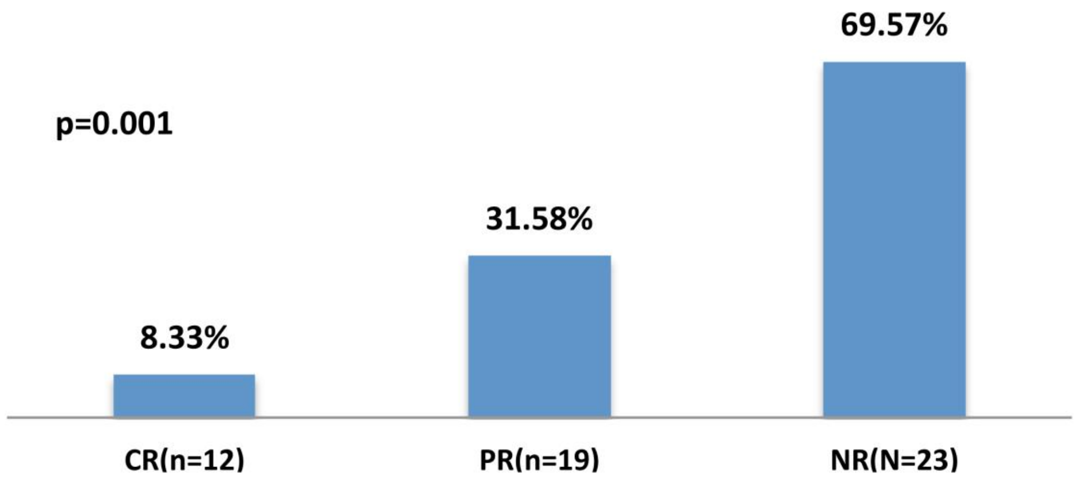

Table S1: Clinical features in MN in cross-sectionally group (n=54)

Clinical features		
Sex (M%)		64.81
Age (year)		50(15-87)
A-PLA2Rab assay from on set (month)		11(1-115)
Treatment	ACEI/ARB	5
	Steroid therapy	2
	Steroid + Immunosuppressent	47
24h-Proteinuria (g/24)		2.45(0.77-15.58)
Albumin (g/l)		27(8-42)
Serum creatinine (umol/l)		45(77-168)
GFR		96(38-165)
